# Improved solid-phase microextraction extraction procedure to detect trace-level epichlorohydrin in municipal water systems by HS-SPME-GC/MS

**DOI:** 10.3389/fchem.2022.1004269

**Published:** 2022-09-27

**Authors:** Ping Lei, Lu Wang, Yun Yan, Wubin Deng, Jingsi Gao, Jia Zhu, Miaoqing Liang, Jiaheng Wen, Jianfeng Lv, Jianfeng Zhou

**Affiliations:** ^1^ Shenzhen Hydrology and Water Quality Center, Shenzhen, Guangdong, China; ^2^ Shenzhen Polytechnic, Shenzhen, Guangdong, China; ^3^ Georgia Tech Shenzhen Institute, Tianjin University (GTSI), Shenzhen, Guangdong, China

**Keywords:** epichlorohydrin, headspace solid phase microextraction, sodium sulfate, drinking water systems, gas chromatography mass spectrometry

## Abstract

Epichlorohydrin (ECH) is toxic to humans *via* multiple routes and is a potential carcinogen. The accurate measurement of ECH at trace level (<0.1 μg/L) is still an obstacle hindering the monitoring and regulation of municipal water systems. In this study, an improved headspace solid-phase microextraction (HS-SPME) procedure is developed and optimized to extract and enrich ECH with high sensitivity, accuracy, and precision. A total 17.4-time enhancement in extraction efficiency is achieved compared with the default condition. Specifically, the AC/PDMS/DVB fiber offered a 4.4-time enhancement comparing with the PDMS/DVB fiber. The effects of different mineral salts in SPME were studied and it was found that an addition of 3 g Na₂SO₄ in the SPME head achieved an additional 3.3-time increase. The pattern how sodium sulfate enhanced ECH extraction by salting out is discussed. The optimization of extraction conditions (pH = 7, 35°C, and 20 min extraction duration) brought another 1.2 times further. Combined with gas chromatography with mass spectrometry, the optimized method exhibits curve linearity in the range of 0.02–1.00 μg/L with an R^2^ of 0.998. The limit of detection, precision, and accuracy of the method are 0.006 μg/L, 2.6%–5.3%, and −3.5% to −2.0%, respectively. The recovery of ECH spiking in tap water and surface water was investigated, with recovery rates of 88.0%–116% and 72.5%–108%, respectively. Adhering to the requirements of existing water quality regulations, our method shows a high potential to be applied in drinking water quality monitoring and water treatment process assessment.

## 1 Introduction

Epichlorohydrin (ECH) is widely used as the chemical precursor in producing glycerol, plastic, epoxy resin, and other chemical products (Hebeish et al., 2011). ECH is toxic to both humans and animals through various contact routes, including inhalation, dermal, oral ingestion, etc. (Currie and Schmieder, 2009; Shin et al., 2010). Acute exposure to a high dose of ECH may cause swollen and blistered tissue, or burning of the eyes and throat through physical contact (Giri, 1997; Ibach et al., 2022). Long-term chronic effects of ECH include heart diseases, genetic toxicity, and potential induction of cancer (Waidyanatha et al., 2014). Therefore, the International Agency for Research on Cancer (IARC) classified ECH as a group 2A substance (probably carcinogenic to humans) (Kolman et al., 2002).

After being released into the environment without proper treatment, ECH might infiltrate municipal water systems and cause pollution (WHO, 2004). As a part of the drinking water quality, ECH is also regulated by different countries/regions. The WHO *Guidelines for Drinking-water Quality* and the Chinese recently published *Standers for drinking water quality* (GB5749-2022) both regulate the concentration of ECH to < 0.4 μg/L. The EU’s drinking water standard *Council Directive 98/83/EC* on the quality of water intended for human consumption imposes a limit of 0.1 μg/L on ECH concentration. US EPA sets the maximum contaminant level goal (MCLG) of zero, and the maximum contaminant level (MCL) is based on the dosage of ECH as a water treatment agent. Therefore, accurate determination of trace ECH is essential for the regulation of such toxic substances in drinking water.

In terms of the extraction of ECH from water samples, liquid-liquid extraction, liquid-solid extraction (adsorption), purge and trap, and headspace solid-phase microextraction (HS-SPME) are frequently performed as pretreatment procedures (Lucentini et al., 2005; Gaca and Wejnerowska, 2006; Lasa et al., 2006; Huang et al., 2013; Onur et al., 2018). Liquid–liquid extraction and liquid–solid extraction have a relatively cumbersome operation and high rate of loss of ECH owing to its low boiling point (117.9°C) (Hidayah and Abidin, 2018). As a pretreatment procedure, purge and trap has the advantage of a large injection volume, but with inefficient aerated stripping of ECH as a drawback (Miermans et al., 2000). In comparison, HS-SPME exhibits higher sensitivity by improving the gaseous concentration in the process (Pawliszyn, 2012; Helin et al., 2015).

After the extraction, gas chromatography (GC) with electron-capture detector (ECD), flame ionization detector (FID), or mass spectrometry (GC/MS) are used as the analytical apparatus (Sarzanini et al., 2000; Bruzzoniti et al., 2004; Ripollés et al., 2009). For example, the German method, *Water Quality-Determination of Epichlorohydrin*, applies the liquid-solid extraction followed by the GC-MS or GC-ECD, which offers a limit of determination of 0.5 μg/L in routine analysis. The Chinese guideline Standard of Water quality examination methods for urban water supply (CJ/T 141-2018) employs liquid-liquid extraction and GC-MS for detection, and the detection limit can be brought down to 0.4 μg/L (which requires at least 200 ml water sample). However, the low recovery rate of 44.0%–78.9% from surface water indicates a large probability of false negatives in detection. In general, the sensitivity of MS and ECD are higher than that of FID (Loda et al., 2011). Previous studies have demonstrated a detection limit of 0.1 μg/L by GC-MS and 0.01 μg/L by GC-ECD (Gaca and Wejnerowska, 2006).

In this work, we aim to improve the measurement sensitivity and lower the detection limit of ECH by optimizing the SPME extraction procedures systematically. The performance of two fiber materials was firstly compared. Subsequently, the impact of mineral salt addition, temperature, pH, and extraction duration in SPME was investigated. Finally, the current method was validated in both tap and surface water with ECH spiking.

## 2 Materials and methods

### 2.1 Chemicals

Sodium nitrate, sodium chloride, magnesium sulfate heptahydrate, copper sulfate pentahydrate, and zinc sulfate heptahydrate were obtained from Thermo-Fisher Scientific (168 Third Avenue, Waltham, MA 02451, United States), and anhydrous sodium sulfate were obtained from Merck (126 East Lincoln Avenue, P.O. Box 2000; Rahway, NJ 07065, United States). The mineral salts were heated at 350°C for 2 h prior to use. A phosphate buffer solution (0.2 mol/L) was prepared by dissolving 4.44 g disodium hydrogen phosphate dodecahydrate (Thermo-Fisher Scientific) and 1.18 g sodium dihydrogen phosphate dihydrate (Merck) in deionized (DI) water (Milli-Q Integral 3 water purification system) with a final pH of 7. Methanol of chromatographic purity was purchased from Thermo-Fisher Scientific.

Two types of SPME fibers, SARR11-DVB-120/20 and SARR11-DVB/CWR120/20, were obtained from Zhida, Guangzhou, China, with the same diameters of 1.1 mm and a length of 2 cm, and a 20 ml headspace bottle with cover. The SARR11-DVB-120/20 fiber was coated with polydimethylsiloxane/divinylbenzene (PDMS/DVB), and the SARR11-DVB/CWR120/20 was coated with activated carbon (AC) in addition to the PDMS/DVB, as a new type SPME fiber with a larger adsorption capacity than the traditional ones.

### 2.2 Standard solutions

The ECH standard solution (1,000 mg/L) was provided by the *Research & Monitor Institute of Environmental Protection, Ministry of Agriculture, China*. The fluorobenzene standard solution (20,000 mg/L) was purchased from AccuStandard (125 Market Street, New Haven, CT 06512, United States) and used for the internal standard.

The stock standard solution of ECH (2 mg/L) was prepared in methanol and stored in darkness at 4–10°C before use. The internal standard stock solution (1 mg/L) was stored under identical conditions. Standard samples were prepared by diluting the stock standard solution with DI water. Samples with concentration of 10 μg/L ECH in DI water were used for optimization experiments performed in triplicate for each condition as parallel measurements, while samples with concentration of 20 μg/L ECH in DI water were used for quantitative analysis. The internal standard solution was diluted to 5 μg/L with DI water. For quantitative analysis, 40 µl of the diluted internal standard was added to each sample, thereby making the final concentration 0.02 μg/L.

### 2.3 Solid-phase microextraction procedure

#### 2.3.1 Comparison of solid-phase microextraction fibers

The two types of SPME fibers were applied to 10 ml ECH samples with an ECH concentration of 10 μg/L. Samples were pre-incubated for 5 min and extracted for 10 min at 45°C, followed by the GC/MS analytical procedures.

#### 2.3.2 Comparison of mineral salts

The AC/PDMS/DVB SPME fiber was used. The headspace bottle was filled with 6 mineral salts separately: NaCl, NaNO₃, Na₂SO₄, MgSO₄, CuSO₄, and ZnSO₄. The dosages of each salt are 0, 1, 2, and 3 g, and the experiments were performed with each salt. No more dosage than 3 g was applied because a higher liquid level stained the fiber. 10 ml of the sample was loaded in bottles. The bottles were capped and shaken vigorously to dissolve the mineral salts. The cells were pre-incubated at 40°C for 5 min. Extraction was performed at 45°C for 10 min under 300 rpm oscillation. Desorption was carried out for 3 min.

#### 2.3.3 Optimization of solid-phase microextraction conditions

Serial temperatures (35, 45, 55, 65, and 75°C) were applied to SPME extraction, and the selected dosage of chosen salt was under pH 7 for 10 min. Under optimized temperature, pH values of 2, 4, 7, 9, and 12 were performed, by adjusting the pH of the samples using HCl or NaOH. Additionally, experiments were performed to study the impact of extraction durations (10, 15, 20, and 25 min). The extraction duration was studied by varying the rexposing time of the fiber in the HS vial. The effects were measured as ECH signal intensity by GC-MS.

### 2.4 Gas chromatography mass spectra conditions

A PAL robotic tool change (RTC) automatic injector provided by *Zhida, Guangzhou, China*, was used with the SPME module and the *in situ* oscillatory extraction device. An *Agilent 8890/5977B GC-MS* was used, which was incorporated with a *DB-624UI* Chromatographic column with dimensions of 30 m × 0.25 mm, and 1.4 μm film thickness.

The split injection port (S/SL) was operated at 1:1 at an injection temperature of 230°C. The following temperature program was set for the column incubator: initiated at 60°C for 3 min, raised to 80°C at 5°C/min increment in 5 min, maintained at 80°C for 1 min, and thereafter raised to 170°C at 15°C/min increment. The transmission line temperature was maintained at 250°C. The column flow rate of helium was set at 1 ml/min. The splitless injection was used for SPME optimization and experiments on protectors.

The ion source temperature was set at 250°C, and the quadrupole temperature was set at 150°C. Selective ion monitoring (SIM) was used, and the parameters were set at 57 m/z (quantitative), 49, and 62 m/z for ECH, whereas 96 m/z (quantitative) and 70 m/z were used for the internal standard. The residence time was set at 100 ms for ECH fragments, and 150 ms for the internal standard. No gain for Electron-Multiplying. The target was identified by the retention time and confirmed by the auxiliary ion ratio. The calibration curve was fitted by linear regression using the ratio of area to concentration.

### 2.5 Method performance

Calibration standards with concentrations (0, 0.02, 0.05, 0.10, 0.20, 0.50, and 1.00 μg/L) were prepared for each analyte. The analyte of the 0.02 μg/L concentration was statistically tested 7 times for standard deviation. The standard deviation was multiplied by 3.143 to obtain the Limit of Detection (LOD) defined by the *Environmental monitoring—Technical guideline on drawing and revising analytical method standards* (HJ 168-2010). The LOQ was estimated by 4 times of the LOD. To verify the precision and accuracy, parallel tests were performed separately (6 times) at 0.05, 0.20, and 0.80 μg/L ECH in pure water.

Water produced by three drinking water treatment plants and water obtained directly from three different water reservoirs in Shenzhen, China, was sampled. Recovery rates of 0.05, 0.20, and 0.80 μg/L ECH spiking in above samples were obtained.

## 3 Results and discussion

### 3.1 Extraction efficiency of different solid-phase microextraction fibers

Both the PDMS/DVB and the AC/PDMS/DVB SPME fibers were tested for the extraction of ECH. Extraction of ECH by the AC/PDMS/DVB fiber brought a signal value of 25,352, which is 4.4 times higher than the signal value of 5,786 by the PDMS/DVB fiber Therefore, the activated carbon serving as the additional coating increased the adsorption efficiency of ECH substantially. The following experiments were carried out with the AC/PDMS/DVB fiber to study its performance and optimize the operational conditions.

### 3.2 Impact of mineral salts on the extraction efficiency

**FIGURE 1:**
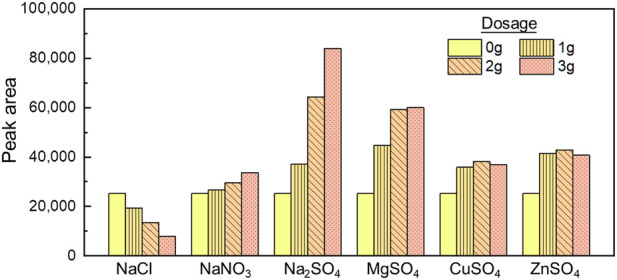
Respond of ECH by SPME-GC/MS with the addition of different mineral salts (pH = 7).

Salting is one of the common methods to enhance the extraction of organics from water samples (Pawliszyn, 2012). Therefore, the impact of six mineral salts of different dosages (0, 1, 2, 3 g) on the extraction by HS-SPME was investigated experimentally. As shown in Figure 1, NaCl inhibited the extraction, as evident by the decreased signal with higher dosage. A dosage of 3 g NaCl reduced 68% of extraction compared with the control group. Such results are inconsistent with previous studies by Lasa (Lasa et al., 2006), who both reported that NaCl helped ECH extraction. On the other hand, the other five salts (NaNO_3_, CuSO_4_, ZnSO_4_, MgSO_4_, Na_2_SO_4_) promoted the ECH extraction. NaNO_3_ slightly promoted the signal with 5%, 16%, and 33% at the dosage of 1, 2, and 3 g, respectively. CuSO_4,_ ZnSO_4,_ and MgSO_4_ brought the highest extraction under 2 g, with an enhancement of 50%, 69%, and 134% than the zero-addition condition. Na₂SO₄ significantly promoted the extraction as a 46% increase at 1 g, 154% increase at 2 g, and 231% increase at 3 g dosage. A dosage of 3 g Na_2_SO_4_ brought the signal to 83,882, which is 3.3 times higher than the zero addition.

When being dissolved in water, the epoxy structure of ECH combines with the hydrogen bond of water molecules, which prevents ECH from being extracted. The charge density of the ions of mineral salts affects the structure of the water, while the majority of positive ions promotes the conversion of water molecules into clusters (weakening matrix effects of hydrogen bonds), and a few negative ions destroy clusters. Both influences are proportional to the molality of ions (Omta et al., 2003). It is speculated that NaCl promoted the high-frequency ionization of water molecules, which made hydrogen ions easy to bond with ECH, resulting in the increase of solubility of ECH and inhibiting ECH extraction efficiency. Other salts with low polarity and electron density help water molecules form clusters and reduce the hydrogen bond activity due to their tetrahedron structure and electronic orbit hybridization. Based on it, sodium sulfate enhanced ECH extraction by increasing the viscosity of the aqueous solution. Similarly, Na^+^ and Mg^2+^ own the higher positive charge density and enhance the self-aggregation of water molecules stronger than Cu^2+^ and Zn^+^.

### 3.3 Optimizing the solid-phase microextraction extraction conditions

#### 3.3.1 Temperature

**FIGURE 2:**
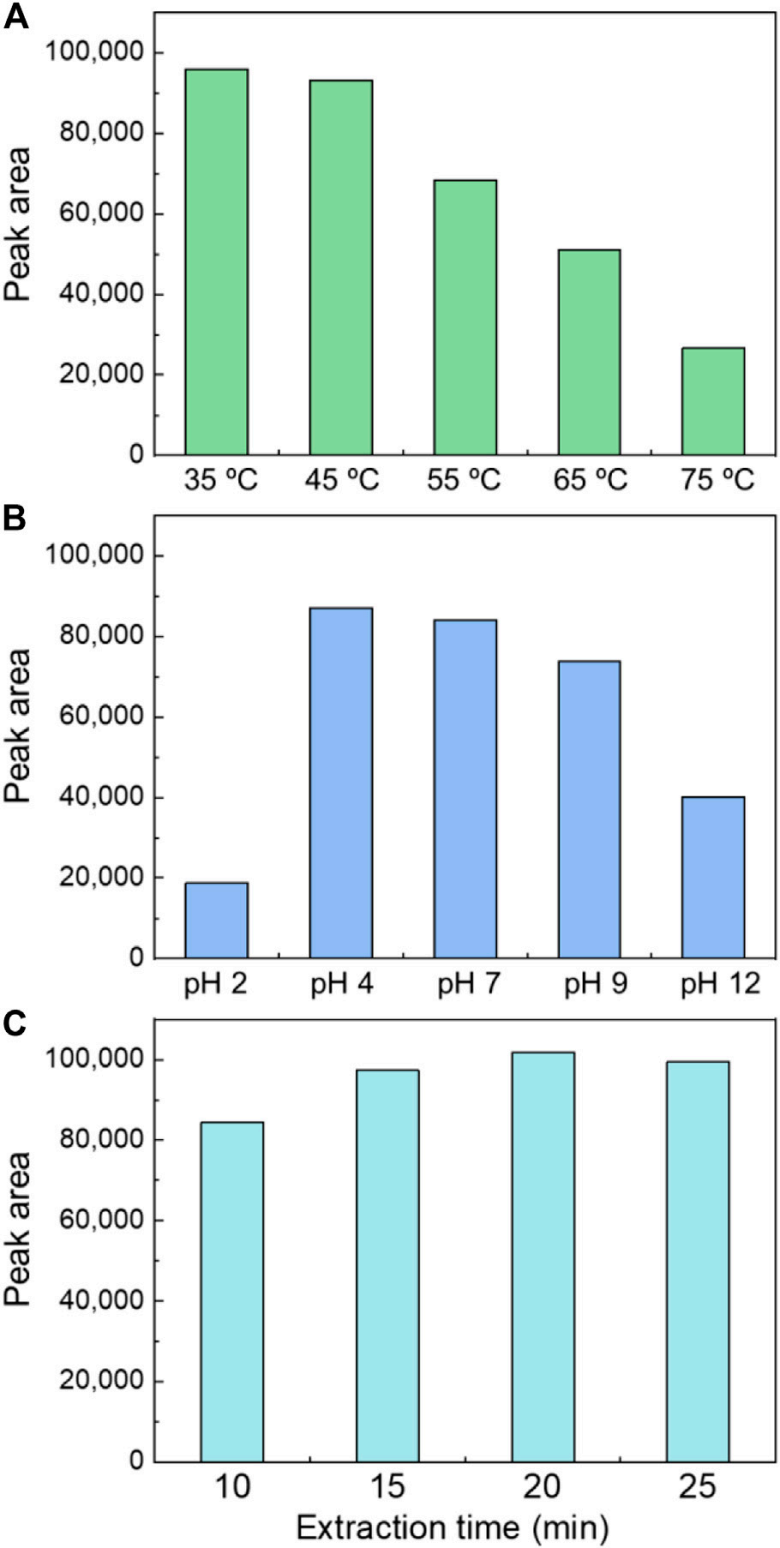
Optimizing the SPME extraction conditions for ECH detection. Impact of **(A)** Temperature, **(B)** pH, and **(C)** Extraction time.

The extraction temperature shows a two-way effect on the extraction efficiency of organics. Higher extraction temperature increases the partitioning of organics to the headspace, while on the other hand, the partitioning into the sorbent may decrease. Therefore, the impact of temperature on the extraction efficiency was tested with the AC/PDMS/DVB fiber and a dosage of 3 g Na₂SO₄. The temperature was controlled at 35, 45, 55, 65, and 75°C. As shown in Figure 2A, increasing the temperature from 35°C to 45°C did not significantly impact the signal. When the temperature further increased to 55, 65, and 75°C, the signal decreased drastically with a percentage of 28, 47, and 73%, respectively. Therefore, when it is higher than 45°C, the temperature is negatively correlated with the extraction concentration. The phenomenon may be caused by influence of temperature on hydro bond. When the temperature increases, there is a tendency that lager clusters formed by water molecules break up because of destruction of hydrogen bond. Therefore, the elevation of temperature significantly decreases the extraction efficiency. However, our result is inconsistent with previous studies. Yu Peizhi and Lu (2018) reported that temperature affected the gas-liquid partition coefficient, and 50°C is the optimal extraction temperature if sodium chloride is not added to the aqueous solution of SPME. On the other hand, Lasa et al. (2006) reported that adding 300 g/L of sodium chloride produced the highest extraction at a low temperature of 5°C. These differences may be due to the two-way effect of the temperature on the partitioning processes.

#### 3.3.2 pH

The effects of different pH (2, 4, 7, 9, and 12) were investigated on SPME under the current optimized conditions (3 g Na₂SO₄ addition with the AC/PDMS/DVB fiber at 35°C). Figure 2B shows that the maximum signal was yielded at pH 4. Signals obtained at pH 2, 9, and 12 decreased by 77%, 12%, and 52% than that at pH 7. No significant difference between pH 4 and pH 7 was observed. It is consistent with a previous study conducted by Guoyong Lu and Lai (2017) who found that the ECH signal faded considerably at pH 1.8, caused by the epoxy structure of ECH. The distribution coefficient of ECH in the water phase is increased under acidic conditions. The lone pairs of oxygen atoms in ECH effectively combine with hydrogen ions (Gutha et al., 2017; Pembere et al., 2017).

### 3.3.3 Extraction duration

The effects of extraction duration (10, 15, 20, and 25 min) were investigated by comparing the signal values. Figure 2C showed that the signal values obtained at 15 and 20 min were 15% and 21% higher than that at 10 min, then slightly reduced to 18% higher at 25 min. This phenomenon is caused by the saturation condition of the fibers. The adsorption of the fiber was not saturated in 10 min, subsequently became fully saturated in 20 min, and supersaturated in 25 min.

### 3.4 Application to natural water samples

So far, the optimal conditions have been obtained: 3 g Na_2_SO₄ addition into the AC/PDMS/DVB fiber at pH 7 and 35°C with a 20 min extraction duration. The signal was increased 17.4 times further in total compared with the default condition. Specifically, the AC/PDMS/DVB fiber, Na₂SO₄ addition, and optimized conditions contributed to the extraction efficiency for 4.4, 3.3, and 1.2 times, respectively.

**FIGURE 3:**
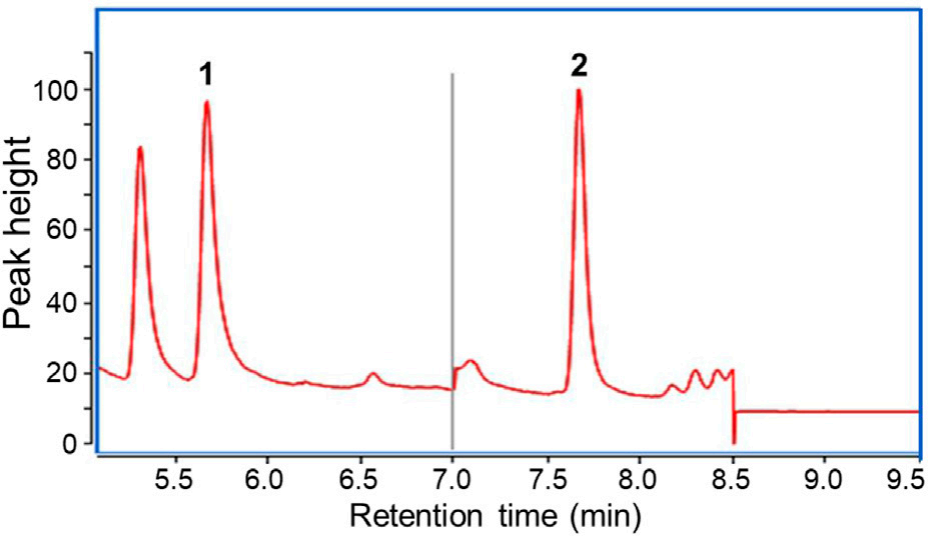
Total Ion Chromatography obtained from the SPME-GC/MS analysis. 1 ppb ECH. Peak 1 indicates the internal standard and peak 2 indicates the ECH.

The GC-MS chromatogram of the water sample is shown in Figure 3. A symmetrical peak of ECH is obtained and labeled with “2”. According to the developed method, the retention time of ECH is 7.672 min, and the retention time of the internal standard is 5.667 min. A calibration curve was constructed (y = 0.0128x−0.0089) with a correlation coefficient (R^2^) of = 0.998. A standard solution with 0.02 μg/L ECH was tested seven times, resulting in a measured range of 0.018–0.021 μg/L. The limit of detection (LOD) is 0.006 μg/L, and the limit of quantitation (LOQ) is 0.024, with 0.02 μg/L as the lowest concentration of the standard curve.

**FIGURE 4:**
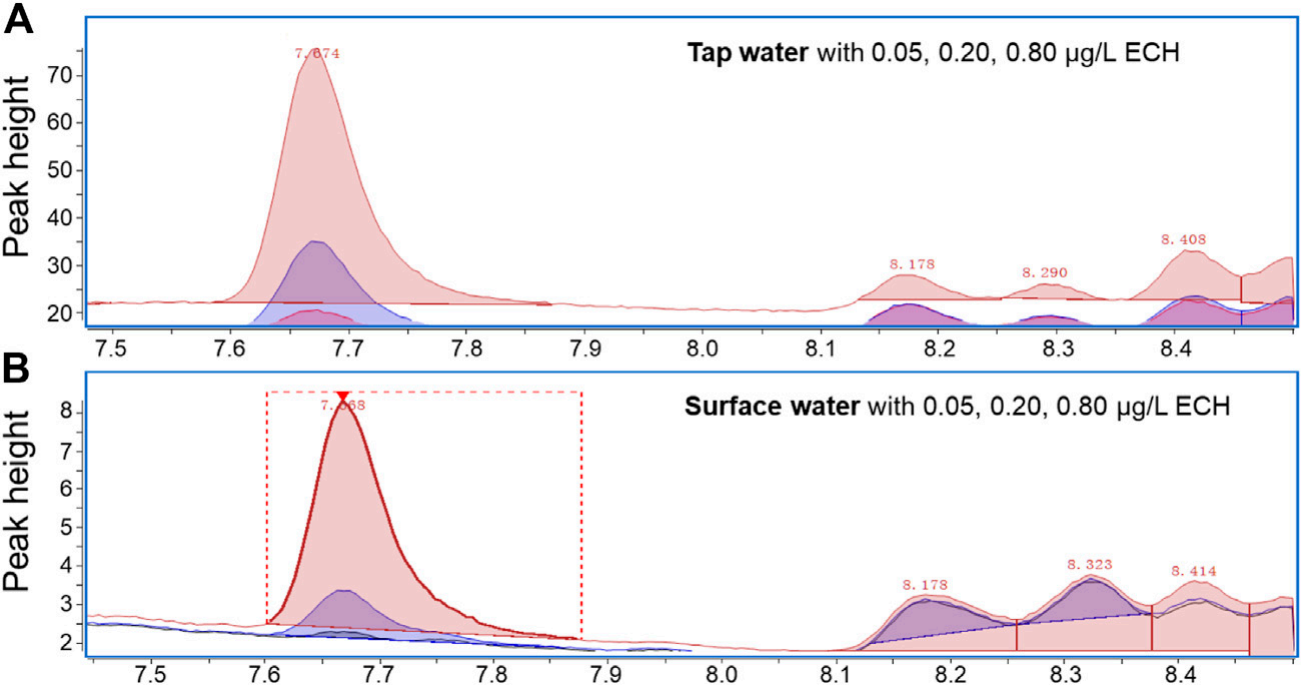
Chromatogram (EIC, m/z = 57) from GC/MS analysis of ECH standard solutions added in **(A)** tap water and **(B)** surface water.

The optimized extraction conditions were used to analyze different water samples (pure, tap, and surface) spiked with ECH. The ECH was not detectable in pure, tap, and surface water samples without spiking. Figure 4 shows the results of spiking in different water matrices, and a symmetrical peak with a detention time of 7.672 min is observed in all samples. Such a distinguishable peak indicates a high sensitivity of the current measurement and optimized conditions. The precision, accuracy, and recovery rate in different water samples with different spiking doses is shown in Table 1. The precision indicates the standard deviation of the data collected, and the accuracy indicates mean absolute error. The method exhibits a precision of 2.6%–5.3% and an accuracy of −3.5% to −2.0% for the ECH standard in pure water. The recovery rate in both tap water (88.0%–116%) and surface water (72.5%–108%) is high and satisfactory, which indicates the advantages of the current study over the previous ones.

**TABLE 1:** The precision, accuracy, and recovery rate of the developed method with different ECH dosages in tap and surface water.

ECH (μg/L)	Precision (%)	Accuracy (%)	Recovery (%)					
			Tap water			Surface water		
			#1	#2	#3	#1	#2	#3
0.05	2.6	−2.0	116	104	88.0	108	100	104
0.20	1.9	−2.0	86.0	90.0	89.5	98.0	72.5	80.5
0.80	5.3	−3.5	96.1	88.2	101	98.8	76.4	82.2

### 3.5 Limitations and future research

Current experimental conditions caused some limitations. Only two available types of SPME fibers were compared in this study, which may cause incompleteness on ECH extraction effect research by SPME. Additionally, the speculated explanation that hydrogen bonds in water clusters affect the extraction efficiency needs more systematic theoretical support. Theoretically, controlling self-aggregation of water molecules or guiding hydrogen to bond other chemicals may dramatically increase the ECH volatility and thus detection sensitivity. Other approaches, such as ionization, deserve further study in the future.

## 4 Conclusion

In this study, a method combining HS-SPME and GC/MS for ECH detection was developed. Adding different mineral salts with different dosages also impacted the measurement differently. The addition of NaCl reduced the extraction efficiency while other salts (NaNO_3_, CuSO_4_, ZnSO_4_, MgSO_4_, Na_2_SO_4_) promoted. It was founded that an addition of 3 g Na₂SO₄ achieved a 3.3-time enhancement in the signal. The operation parameters were optimized to a pH of 7, a temperature of 35°C, and an extraction time of 20 min. Such condition optimization yielded a 1.2-time enhancement, and thus the overall enhancement, including the fiber selection, salting, and condition optimization is 17.4 times of the default setting. Measurements of tap and surface water with ECH spiking were conducted, and a low detection limit (0.006 μg/L) was observed.

## Data Availability

The raw data supporting the conclusion of this article will be made available by the authors, without undue reservation.
